# LINC00052 ameliorates acute kidney injury by sponging miR-532-3p and activating the Wnt signaling pathway

**DOI:** 10.18632/aging.104152

**Published:** 2020-11-24

**Authors:** Xiaoying Li, Pengxi Zheng, Tingting Ji, Bo Tang, Yakun Wang, Shoujun Bai

**Affiliations:** 1 Department of Nephrology, Qingpu Branch of Zhongshan Hospital Affiliated to Fudan University, Qingpu 201700, Shanghai, P.R. China

**Keywords:** acute kidney injury, LINC00052, miR-532-3p, Wnt signaling

## Abstract

Acute kidney injury (AKI) is a complex renal disease. Long non-coding RNAs (lncRNAs) have frequently been associated with AKI. In the present study, we aimed to investigate the molecular mechanism(s) of LINC00052 in AKI. We found that LINC00052 expression was significantly decreased in AKI patient serum. In addition, in a hypoxic AKI cell model, LINC00052 expression was strongly elevated. In an I/R-triggered AKI rat model, the expression of TNF-α, IL-6 and IL-1β mRNA was strongly elevated. Moreover, we predicted miR-532-3p to be targeted by LINC00052 in AKI. Overexpression of LINC00052 increased hypoxia-induced inhibition of NRK-52E cell proliferation and reversed hypoxia-triggered apoptosis. Furthermore, we found that induction of TNF-α, IL-6 and IL-1β was repressed by overexpression of LINC00052. LINC00052 decreased hypoxia-induced ROS and MDA accumulation *in vitro* and increased SOD activity. Decreased levels of c-myc and cyclin D1 were observed in renal tissues of AKI rats. Lastly, Wnt/β-catenin signaling was inactivated in NRK-52E cells experiencing hypoxia, and LINC00052 upregulation reactivated Wnt/β-catenin signaling by sponging miR-532-3p. Taken together, these results suggest that LINC00052 ameliorates AKI by sponging miR-532-3p and activating Wnt signaling.

## INTRODUCTION

Acute kidney injury (AKI) is a serious syndrome associated with renal dysfunction. AKI is a frequent complication of surgical operations that results in loss of kidney function [1, 2], and is associated with long-term hospital stay, chronic kidney disease and end-stage renal disease [3, 4]. Acute vascular dysfunction, tubular epithelial cell injury, inflammation, and fibrosis contribute to AKI pathophysiology [5].

Ischemia-reperfusion (I/R) injury is a known cause of AKI with high morbidity and mortality [6, 7]. Inflammation can mediate tissue repair after renal injury and participates in the development of IR-triggered AKI [8]. Additionally, oxidative stress has been linked to AKI [9]. Under normal conditions, the production of oxidants and antioxidants remains in equilibrium. However, oxidative stress can arise when this balance is disrupted [10].

Long non-coding RNAs (lncRNAs) are RNA molecules that are over 200 nucleotides in length. lncRNAs play critical roles in a variety of biological processes [11–13]. In recent years, many lncRNAs have been linked to renal disease [14]. For example, downregulation of the MEG3 lncRNA protects against kidney injury induced by hypoxia in renal allografts by modulating miR-181b and targeting TNF-α [15]. The ZFAS1 lncRNA can promote clear cell renal cell tumor progression by regulating miR-10a and SKA1 [16]. In addition, the NEAT1 lncRNA can promote extracellular matrix accumulation and the epithelial-mesenchymal transition by modulating miR-27b-3p and ZEB1 in diabetic nephropathy [17]. Loss of the TUG1 lncRNA results in the development of AKI by sponging miR-142-3p and regulating the sirtuin 1 axis [18].

In this study, we report that LINC00052 expression is strongly decreased whereas miR-532-3p expression is increased in AKI. Overexpression of LINC00052 inhibits hypoxia-induced injury of NRK-52E cells by activating the Wnt/β-catenin signaling pathway.

## RESULTS

### LINC00052 expression is reduced while miR-532-3p expression is elevated in AKI patients and NRK-52E cells experiencing hypoxia

**Figure 1:**
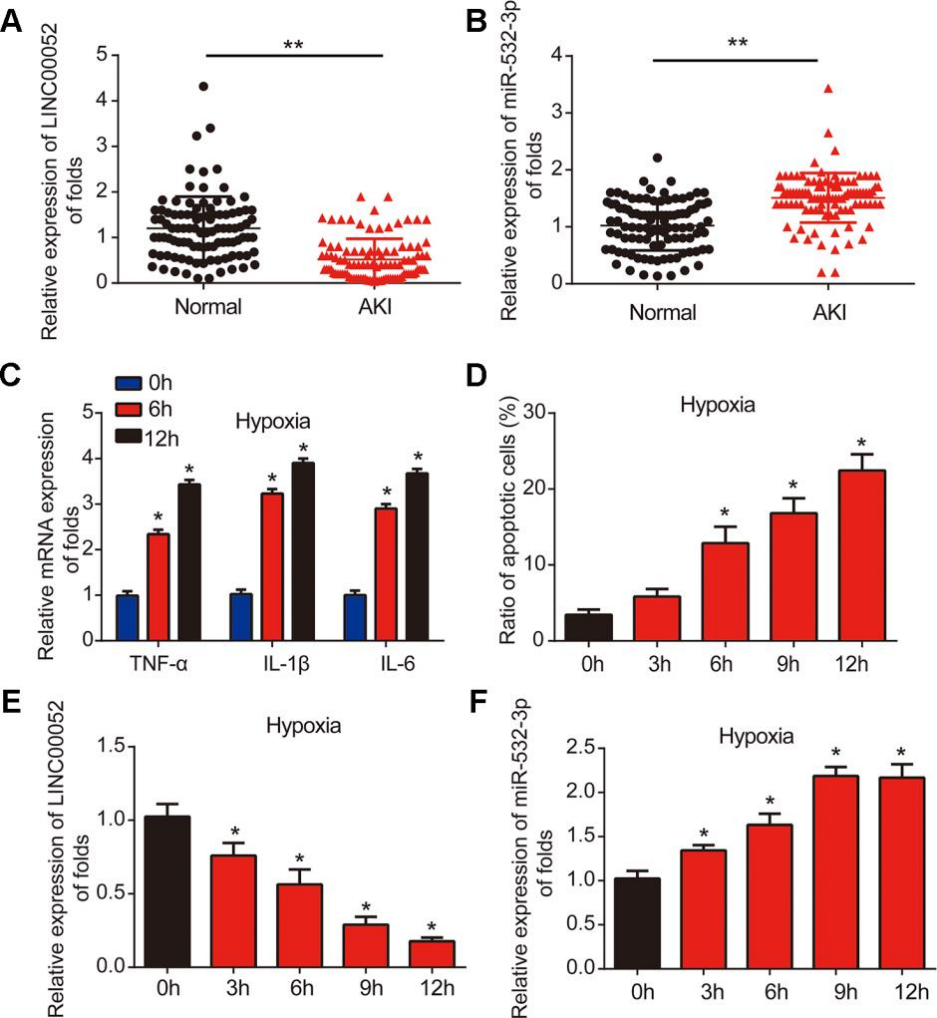
**LINC00052 is downregulated miR-532-3p is upregulated in AKI patient tissues and AKI cell models.** (**A**) Analysis of LINC00052 expression in serum from healthy controls (*n* = 100) and AKI patients (*n* = 100). GAPDH served as a loading control. (**B**) miR-532-3p expression in serum from healthy controls (*n* = 100) and AKI patients (*n* = 100). U6 served as a loading control. (**C**) The expression of IL-1β, IL-6 and TNF-α mRNAs in NRK-52E cells exposed to hypoxia. (**D**) Apoptosis of NRK-52E cells was measured by flow cytometry. NRK-52E cells were exposed to a hypoxia time-course. (**E**) Analysis of LINC00052 expression in NRK-52E cells after 0, 3 h, 6 h, 9 h or 12 h of hypoxia. (**F**) Analysis of miR-532-3p expression in NRK-52E cells after 0, 3 h, 6 h, 9 h and 12 h of hypoxia. Three independent experiments were performed. Error bars represent the mean ± SD of triplicate experiments (at least). *p < 0.05; **p < 0.01.

Firstly, we measured the levels of LINC00052 and miR-532-3p expression in serum from AKI patients (*n* = 100) and healthy controls (*n* = 100) using qRT-PCR. The level of LINC00052 was decreased in AKI patient serum (Figure 1A), while the level of miR-532-3p was increased in AKI patient serum (Figure 1B). Next, we developed a hypoxia-induced NRK-52E cell model. We found that the expression of TNF-α, IL-1β and IL-6 mRNA was induced by hypoxia after 6 and 12 h (Figure 1C). Flow cytometry assays demonstrated that apoptosis was triggered in NRK-52E cells in a time-dependent manner starting after 6 h of hypoxia (Figure 1D). We then uncovered that LINC00052 expression was decreased in hypoxic NRK-52E cells (Figure 1E), while miR-532-3p expression was strongly upregulated in hypoxic NRK-52E cells (Figure 1F). These results suggest that LINC00052 and miR-532-3p might be involved in AKI.

### Analysis of LINC00052 and miR-532-3p expression in AKI rat models

**Figure 2:**
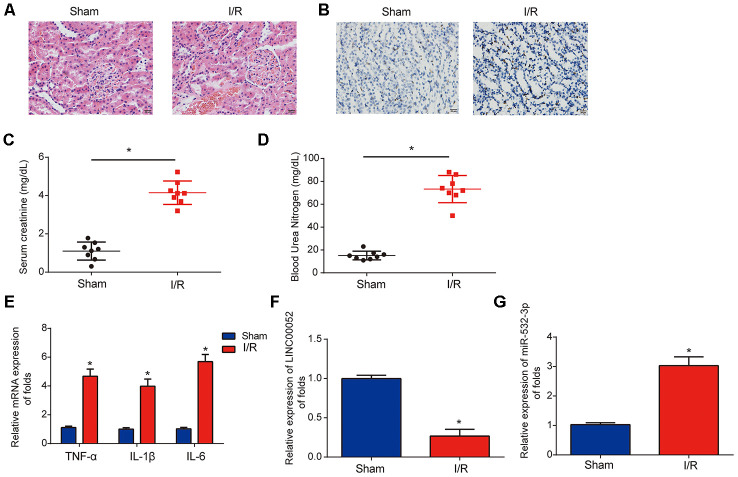
**Analysis of LINC00052 and miR-532-3p expression in AKI rat models triggered by I/R surgery.** (**A**) Renal histology micrographs of renal tissues from I/R-induced AKI rat models. Scale bars = 20 μm. (**B**) TUNEL assays measuring apoptosis in renal tissues from I/R-induced AKI rat models. Serum levels of SCr (**C**) and BUN (**D**) in renal tissues from I/R-induced AKI rats 24 h after surgery. (**E**) The expression of IL-1β, IL-6 and TNF-α mRNAs in renal tissues from I/R-induced AKI rats 24 h after surgery was assessed by qRT-PCR. Analysis of LINC00052 (**F**) and miR-532-3p (**G**) expression in renal tissues from I/R-induced AKI rats using qRT-PCR. Three independent experiments were performed (*n* = 8 in each group). Error bars represent the mean ± SD of triplicate experiments (at least). *p < 0.05.

Next, we established I/R-triggered AKI rat models to explore the roles of LINC00052 and miR-532-3p in AKI. We confirmed induction of acute kidney injury through HE staining and TUNEL assays (Figure 2A and 2B). We observed that SCr and BUN were strongly upregulated in the serum of AKI rat models 24 h after I/R surgery (Figure 2C and 2D). In addition, we found that TNF-α, IL-1β and IL-6 were induced in renal tissue from AKI rats (Figure 2E). Furthermore, LINC00052 expression was decreased while miR-532-3p expression was increased in the kidneys of AKI rats (Figure 2F and 2G). These results suggest that LINC00052 and miR-532-3p are involved in AKI.

### miR-532-3p is predicted as a downstream target of LINC00052

**Figure 3:**
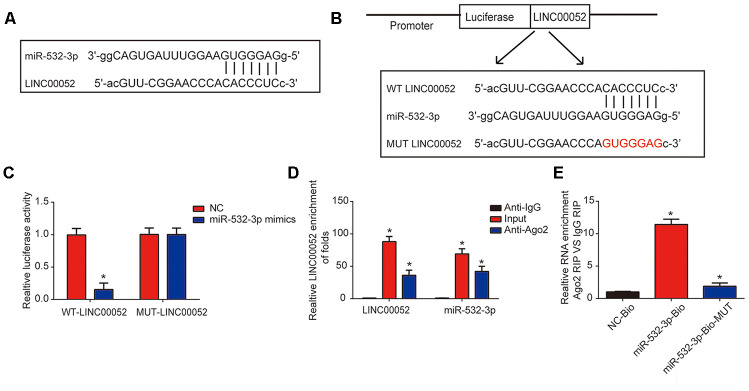
**miR-532-3p is a target of LINC00052.** (**A**) A schematic of the interaction between LINC00052 and miR-532-3p. (**B**) The luciferase reporter constructs containing the wild type (WT-LINC00052) or mutant LINC00052 (MUT-LINC00052). (**C**) The relationship between LINC00052 and miR-532-3p was assessed using a dual luciferase reporter assay. WT-LINC0005 or MUT-LINC00052 were co-transfected into NRK-52E cells with miR-532-3p mimics or their corresponding negative controls. (**D**) The interaction between LINC00052 and miR-532-3p was assessed using a RIP assay. (**E**) RNA pull-down assays detected a direct interaction between LINC00052 and miR-532-3p. Three independent experiments were performed. Error bars represent the mean ± SD of triplicate experiments (at least). *p < 0.05.

The interaction between miR-532-3p and LINC00052 is illustrated in Figure 3A. Luciferase WT-LINC00052 and MUT-LINC00052 reporter plasmids were constructed (Figure 3B). Inhibited reporter activity was observed upon transfection of WT-LINC00052 and miR-532-3p mimics (Figure 3C). To determine whether LINC00052 sponged miR-532-3p, RIP experiments were performed. miR-532-3p and LINC00052 were enriched in Ago2 pellet (Figure 3D). RNA pull-down assays using a miR-532-3p-bio probe produced a higher level of LINC00052 than pull-downs with negative control-bio or miR-532-3p probes (Figure 3E). These results indicate that miR-532-3p is a target of LINC00052.

### The effects of LINC00052 on proliferation and apoptosis of NRK-52E cells are reversed by miR-532-3p

**Figure 4:**
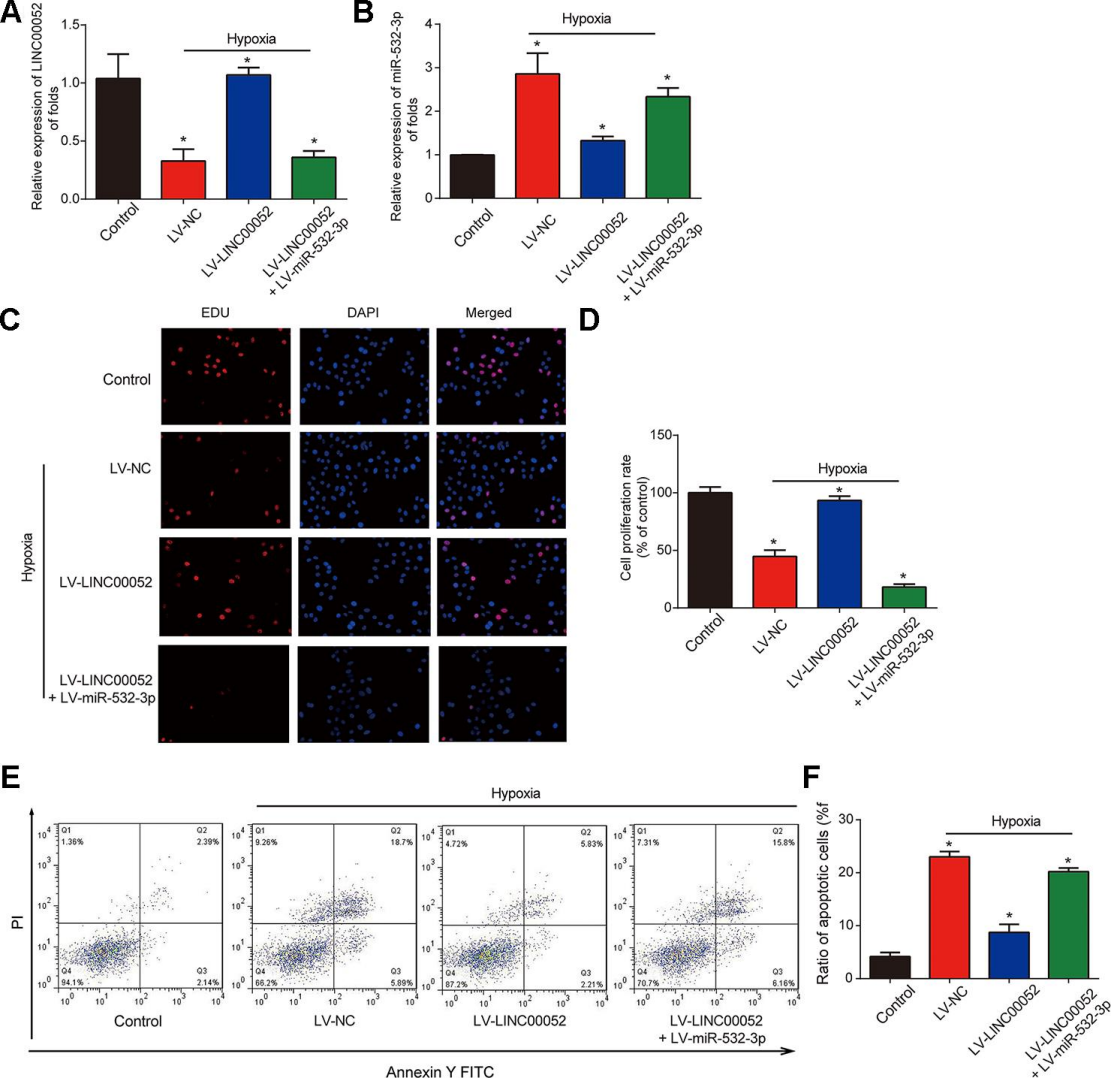
**The impacts of LINC00520 and miR-532-3p on NRK-52E cells experiencing hypoxia.** (**A**) LINC00052 expression in NRK-52E cells infected with LV-LINC00052 or LV-LINC00052 and LV-miR-532-3p under hypoxic conditions. (**B**) miR-532-3p expression in NRK-52E cells infected with LV-LINC00052 or LV-LINC00052 and LV-miR-532-3p. (**C** and **D**) NRK-52E cell proliferation was detected using an EdU assay. Cells were infected with a combination of LV-LINC00052 and LV-miR-532-3p or LV-LINC00052 and LV-miR-532-3p. (**E** and **F**) NRK-52E cell apoptosis was measured using flow cytometry. Three independent experiments were performed. Error bars represent the mean ± SD of triplicate experiments (at least). *p < 0.05.

NRK-52E cells were infected with LV-LINC00052 and LV-miR-532-3p under hypoxia. We found that LINC00052 expression was elevated in NRK-52E cells (Figure 4A). Meanwhile, we found that miR-532-3p expression was repressed by LV-LINC00052 and this was reversed by LV-miR-532-3p (Figure 4B). Meanwhile, overexpression of LINC00052 rescued the reduction in cell proliferation, while overexpression of miR-532-3p exhibited the opposite effect (Figure 4C and 4D). Hypoxia-induced apoptosis was repressed by LV-LINC00052 overexpression in NRK-52E cells (Figure 4E and 4F). In contrast, miR-532-3p overexpression increased apoptosis, an effect which was inhibited by LINC00052. These results indicate that LINC00052 increases NRK-52E cell proliferation and represses apoptosis by sponging miR-532-3p.

### The effects of LINC00520 on inflammation and ROS levels in hypoxic NRK-52E cells are rescued by miR-532-3p

**Figure 5:**
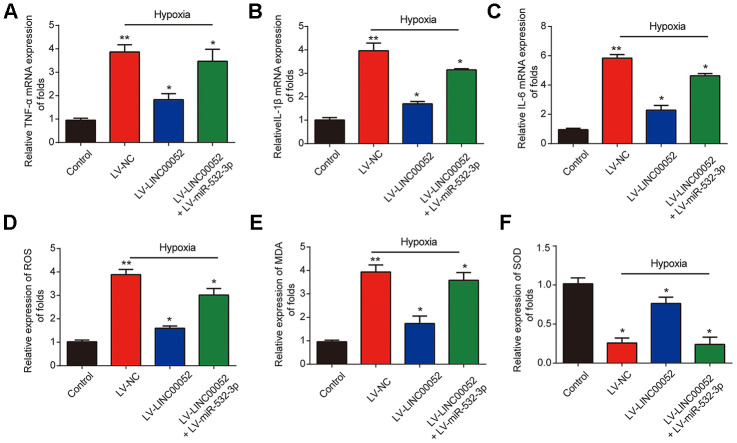
**The impacts of LINC00520 and miR-532-3p on inflammation and ROS levels in NRK-52E cells experiencing hypoxia.** Expression of TNF-α (**A**), IL-1β (**B**) and IL-6 (**C**) mRNAs in NRK-52E cells infected with a combination of LV-LINC00052 and LV-miR-532-3p or LV-LINC00052 and LV-miR-532-3p was tested using q-RT-PCR. The levels of ROS (**D**), MDA (**E**), and SOD (**F**) under different conditions. Three independent experiments were performed. Error bars represent the mean ± SD of triplicate experiments (at least). *p < 0.05.

Furthermore, we measured the expression of TNF-α, IL-1β and IL-6 mRNA in NRK-52E cells infected with a combination of LV-LINC00052 and LV-miR-532-3p or LV-LINC00052 and LV-miR-532-3p using qRT-PCR. LINC00052 repressed inflammation in NRK-52E cells (Figure 5A–5C). TNF-α, IL-1β and IL-6 were repressed by LINC00052 and induced by miR-532-3p. The increases in ROS and MDA levels observed in NRK-52E cells were repressed by LINC00052 and further exacerbated by miR-532-3p, whereas SOD exhibited the opposite effect (Figure 5D–5F). These results suggest that LINC00052 affects inflammation and ROS levels in NRK-52E cells by modulating miR-532-3p.

### miR-532-3p abolishes the inhibitory effect of LINC00520 in NRK-52E cells by inactivation of the Wnt signaling pathway

**Figure 6:**
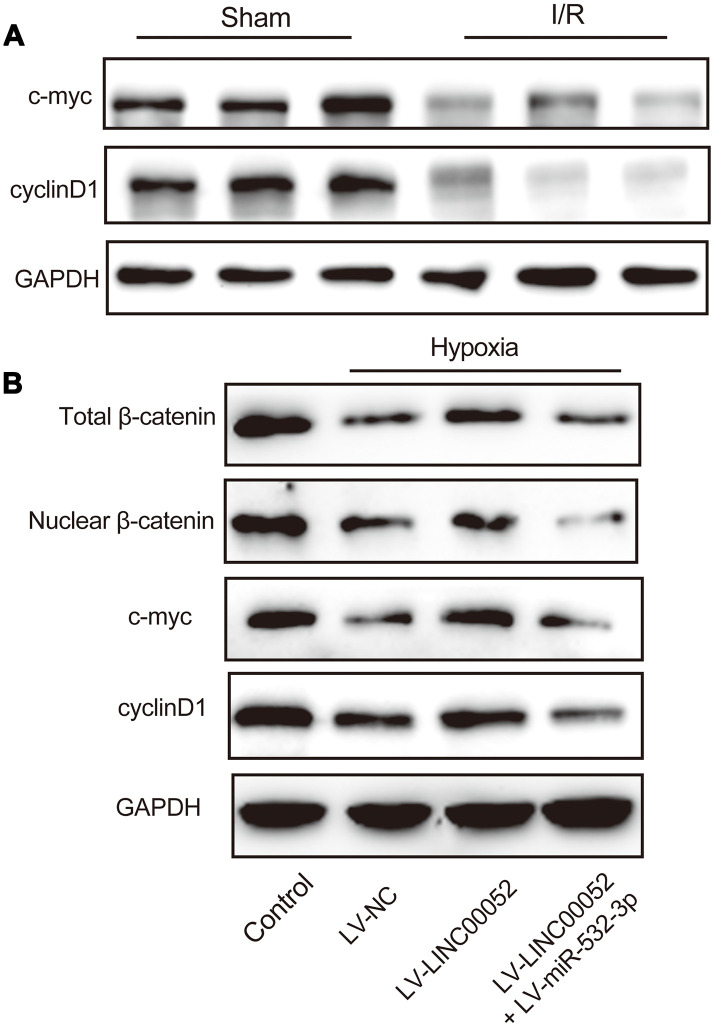
**miR-532-3p abolishes the repressive effect of LINC00520 by inactivation of the Wnt/β-catenin pathway in NRK-52E cells.** (**A**) IHC staining of c-myc and cyclin D1 in AKI rat models. (**B**) Expression of β-catenin, c-myc, and cyclin D1 proteins in NRK-52E cells infected with a combination of LV-LINC00052 and LV-miR-532-3p or LV-LINC00052 and LV-miR-532-3p. Three independent experiments were performed. Error bars represent the mean ± SD of triplicate experiments (at least). *p < 0.05.

Inactivation of the Wnt/β-catenin pathway has been linked to AKI progression. We observed that levels of c-myc and cyclin D1 were decreased in renal tissue of AKI rats through IHC staining (Figure 6A). Subsequently, we found that hypoxic NRK-52E cells exhibit inactivation of Wnt/β-catenin signaling, and this signaling pathway was reactivated by LINC00052 by sponging miR-532-3p. β-catenin, c-myc and cyclin D1 protein expression was upregulated by LINC00052 and downregulated by miR-532-3p (Figure 6B). These results indicate that inactivation of Wnt/β-catenin pathway is involved in LINC00052-mediated AKI progression.

## DISCUSSION

In recent years, an increasing number of lncRNAs and microRNAs have been associated with AKI [19–21]. For instance, the NEAT1 lncRNA induces hypoxia-triggered renal tubular epithelial cell apoptosis by sponging miR-27a-3p [22]. miR-27b-3p targeted by LINC00520 regulates OSMR expression and induces AKI development via the PI3K/AKT signaling pathway [23]. In HK-2 cells, the PVT1 lncRNA can promote LPS-triggered septic AKI by modulating TNF-α and JNK/NF-κB [24].

We investigated the biological roles of LINC00052 and miR-532-3p in AKI. LINC00052 expression was reduced while miR-532-3p expression was up-regulated in AKI patient serum, hypoxic NRK-52E cells and AKI rat models. LINC00052 overexpression reversed the effects of hypoxia on NRK-52E cell proliferation and apoptosis by sponging miR-532-3p. In addition, we observed that the Wnt/β-catenin pathway was inactivated in hypoxic NRK-52E cells, and miR-532-3p overexpression abolished the inhibitory effect of LINC00520 by inactivating Wnt signaling.

LINC00052 has been recognized as a crucial regulator of tumor processes. For example, LINC00052 increases EPB41L3 to repress hepatocellular carcinoma migration and invasion by binding miR-452-5p [25]. LINC00052 represses the proliferation, migration and invasion of glioma cells by upregulating KLF6 [26]. LINC00052 promotes gastric cancer progression by activating the Wnt signaling pathway [27]. Here, we found that LINC00052 was downregulated in AKI. Increasing LINC00052 expression induced NRK-52E cell proliferation and inhibited apoptosis. These results indicate that LINC00052 plays a significant role in AKI progression.

We predicted that miR-532-3p was a target of LINC00052. Recently, aberrant expression of miR-532-3p has been reported in multiple cancers. miR-532-3p promotes hepatocellular carcinoma progression by targeting PTPRT [28]. miR-532-3p is targeted by DARS-AS1 and functions as an oncogene in ovarian cancer [29]. A role for miR-532-3p in the regulation of renal cell carcinoma has been identified [30]. miR-532-3p is downregulated in progressive chronic kidney disease [31]. In the present study, we focused on the biological role of miR-532-3p in AKI progression. We found that miR-532-3p was upregulated in AKI patients, AKI cells and rat models. miR-532-3p was negatively regulated by LINC00052, and miR-532-3p reversed the inhibitory effect of LINC00052 *in vitro* in AKI.

The Wnt/β-catenin signaling pathway is implicated in the modulation of various biological processes, such as AKI [32, 33]. The Wnt/β-catenin pathway is reactivated after kidney injury to repair fibrotic renal lesions [34]. Increasing evidence suggests that Wnt signaling plays a significant role in improving kidney repair and regeneration after I/R-induced AKI [35]. miR-214 can ameliorate AKI by targeting DKK3 and activating the Wnt pathway [36]. Inhibition of miR-155 inhibits AKI by regulating TCF4 and Wnt/β-catenin [37]. In addition, miR-182 enhances AKI by promoting apoptosis and the TCF7L2/Wnt/β-catenin pathway [38]. We found that the Wnt/β-catenin pathway was inactivated in AKI rat models. Overexpression of LINC00052 reactivated the Wnt/β-catenin pathway in NRK-52E cells by sponging miR-532-3p. In addition, Wnt pathway-associated genes including Cyclin D1, MYCN, and GSK-3B are predicted to be downstream targets of miR-532-3p. Our future work will aim to follow up on these observations.

In conclusion, we report that LINC00052 ameliorates AKI progression by sponging miR-532-3p and activating the Wnt/β-catenin signaling pathway, suggesting that LINC00052 might be a novel therapeutic target to treat AKI.

## MATERIALS AND METHODS

### Clinical samples

Blood samples from 100 AKI patients were obtained from the Qingpu Branch of Zhongshan Hospital Affiliated with Fudan University, and 100 healthy volunteers were recruited as controls. The blood samples collected from AKI patients were centrifuged at 5,000×*g* for 3 min and then serum was harvested. Prior to starting experiments, written informed consent was obtained from all participants. Procedures used in this study were approved by the Institutional Ethics Committee of the Qingpu Branch of Zhongshan Hospital. The experiments were performed according to government policies and the Helsinki Declaration.

### Cells

NRK-52E cells were purchased from ATCC (Manassas, VA, USA). Cells were cultured in DMEM medium (Sigma-Aldrich, St. Louis, MO, USA) supplemented with 10% FBS, 0.15% sodium bicarbonate, 4 mM L-glutamine, and 1% streptomycin/penicillin (Invitrogen, CA, Carlsbad, USA) in an atmosphere with 5% CO_2_ at 37° C. For assays involving hypoxia, cells were maintained in a hypoxic chamber containing 1% O_2_ for 24 h, followed by reoxygenation.

### Construction of rat AKI models

Sixteen male SD rats (280-300 g and 12 weeks) were purchased from the Shanghai Animal Laboratory Center. To generate AKI rat models, rats were anesthetized by intraperitoneal injection of 50 mg/kg pentobarbital sodium. Bilateral renal pedicles were occluded using a microvascular clamp to induce renal ischemia. The clamps were then isolated to trigger reperfusion. Identical surgical procedures were performed on rats with unclamped renal pedicles (sham group). Each group consisted of eight rats. We collected blood, urine, and kidney tissues for analysis. We extracted venous blood (5 mL) from the jugular vein. After centrifugation, we stored the supernatant at -80° C for serum index measurements. Rats were maintained according to the Guide for the Care and Use of Laboratory Animals from the National Academy of Sciences.

### Assessment of renal function

Blood was centrifuged at 3,000 rpm for 10 min at 4° C. We measured the levels of serum creatinine (SCr) and blood urea nitrogen (BUN) as crucial indices of renal injury. Serum creatinine and BUN were detected using detection kits (Nanjing Jian Cheng Institute of Biotechnology, Nanjing, China).

### Kidney tissue histology

To observe pathological changes, kidneys were fixed with 4% (w/v) paraformaldehyde at 4° C for 24 h. Then, we embedded the tissues in paraffin and cut them into 4-μm sections for Hematoxylin and Eosin (H&E) staining (Beijing Solarbio Science and Technology Co., Ltd., Beijing, China). Subsequently, we examined and photographed pathological changes using light microscopy (Olympus America, Inc., NY, U.S.A.).

### IHC staining of c-myc and cyclin D1

Thick sections (4-μm) of the kidney tissues were mounted to slides that were then deparaffinized and rehydrated. The sections were treated with citrate buffer for 30 min. The sections were then immersed in PBS with 3% H_2_O_2_ for 10 min. Rabbit polyclonal anti- c-myc and cyclin D1 antibodies (dilution 1:100, Abcam, Cambridge, UK) were used to block the sections at 4° C for a whole night.

### Lentiviral infection

LV-LINC00052, LV-miR-532-3p and their corresponding negative controls were constructed by GenePharma (Shanghai, China). LV-LINC00052 and LV-miR-532-3p were sub-cloned into lentiviral plasmids and used in conjunction with lentiviral packaging plasmids to infect HEK-293T cells. Cells were infected with LV-LINC00052, LV-miR-532-3p and 8 μg/mL polybrene at an MOI of 3. Blasticidin (10 μg/mL) (Thermo Fisher) was used to select positive cells.

### Evaluation of ROS, MDA and SOD levels

ROS levels were measured using the DCHF-DA fluorescent probe (Jiancheng Biotech, Nanjing, China). Cells at a density of 1 × l0^4^ cells/mL were seeded into 6-well plates. Then, the culture medium was removed, and cells were washed with D-Hank’s solution three times. Cells were then incubated with 10 μM dichloro-dihdro-fluorescence diacetate (DCFH-DA) for 30 min at 37° C. The fluorescent intensity was measured with a fluorescence spectrophotometer. MDA and SOD were measured using the Lipid Peroxidation MDA Assay Kit and Total SOD Assay Kit (Beyotime, Jiangsu, China), respectively. In brief, cells were lysed using 0.2% Triton X-100 and centrifuged at 10,000×*g* for 10 min at 4° C. The supernatant was then transferred to a fresh flat-bottom 96-well culture plate. Further enzymatic analysis was performed according to the manufacturer’s instructions.

### EdU assay

The EdU detection kit (RiboBio, Guangzhou, PR, China) was used to measure cell proliferation. Cells were treated with 50 μM EdU and fixed with 4% paraformaldehyde. Anti-EdU working reagents (0.5%) were used to label the cells. Triton X-100 and 5 μg/mL Hoechst33342 were used to wash the cells and visualize DNA.

### Flow cytometry

Flow cytometry was used to detect cell apoptosis using the Annexin V-FITC apoptosis detection kit (Biolegend, San Diego, CA, USA). After 48 h of transfection, cells were washed with ice-cold PBS. Cells were suspended in 100 μL binding buffer with 5 μL Annexin V-FITC and 5 μL propidium iodide. Apoptosis rates were measured using flow cytometry (BD Biosciences, Franklin Lakes, NJ, USA).

### TUNEL assays

TUNEL assays were performed using the TUNEL detection kit (Promega, Madison, WI, USA). Proteinase K (20 μg/mL) was used as a blocking agent. The sections were incubated with TUNEL mixture for 1 h and HRP-conjugated streptavidin for 30 min. DAB (0.04%) and H_2_O_2_ (0.03%) were utilized for visualization.

### qRT-PCR

Total RNA was isolated using TRIzol reagent (TaKaRa, Tokyo, Japan). Total RNA was reverse-transcribed into DNA using the TaqMan MicroRNA Reverse Transcription kit (Applied Biosystems; Thermo Fisher Scientific, Inc). The TaqMan MicroRNA qPCR assay kit was used to test miR-532-3p expression, and M-MLV (Promega, Madison, WI, USA) was used for cDNA synthesis. cDNA was subjected to quantitative PCR using SYBR Premix Ex Taq™ Kit (TaKaRa, Tokyo, Japan) and an ABI 7900 Fast Real-time PCR system in order to test the expression of LINC00052, TNF-α, IL-6 and IL-1β. Relative gene expression was measured using the 2^−ΔΔCt^ method. The primers used in this study are listed in Table 1.

**Table 1:** Primers used for real-time PCR.

**Genes**	**Forward (5’-3’)**	**Reverse (5’-3’)**
GAPDH	CAAGGTCATCCATGACAACTTTG	GTCCACCACCCTGTTGCTGTAG
U6	CTCGCTTCGGCAGCACA	AACGCTTCACGAATTTGCGT
LINC00052	CCTGAAGTTTCTCCATGAATTGTG	GAGGGAGGGAGACTGAGATT
miR-532-3p	TGATGAGCATCTGAAGACGGA	GGAGGCACAAGGAAAGACCAA
IL-1β	GGATAACGAGGCTTATGTGCACG	GGACATGGAGAACACCACTTGTTG
IL-6	GACTGATGTTGTTGACAGCCACTGC	AGCCACTCCTTCTGTGACTCTAACT
TNF-α	CATGATCCGAGATGTGGAACTGGC	CTGGCTCAGCCACTCCAGC

### Western blot

An equal amount of total protein was separated using 10% SDS-PAGE gels and then transferred to PVDF membranes. Next, membranes were blocked for 2 h in 5% skimmed milk. After overnight incubation at 4° C with primary antibodies (β-catenin, c-myc, cyclin D1 and GAPDH; 1:1000; Abcam, Cambridge, UK), the membranes were washed using TBST. Finally, the ECL Western Blotting Analysis System (GE Healthcare, Chicago, IL, USA) was used to develop signals.

### Luciferase reporter assays

The LINC00052 3′-untranslated region (3’UTR) was cloned into the pGL3-Basic vector (Promega, Madison, WI, U.S.A.). Mutant OXSR1-3′-UTR (MUT) was also cloned into the pGL3 luciferase vector (Promega). Before transfection, cells were seeded into 12-well plates (4 × 10^5^ cells/well) in an atmosphere with 5% CO_2_ at 37°C. To evaluate miR-532-3p target sites in the LINC00052 3’UTR, cells were co-transfected with the wild-type or mutated LINC00052 3’UTR reporter plasmids and miR-532-3p mimics using Lipofectamine 3000 reagent (Invitrogen, San Diego, CA, USA). Both Firefly and Renilla luciferase activities were tested 24 h after transfection using the Dual-Luciferase Reporter Assay System kit ((Promega, Madison, WI, USA).

### RNA immunoprecipitation (RIP)

An Ago2 antibody (Cell Signaling, Danvers, MA, USA) and the Magna RIP RNA-Binding Protein IP Kit (Millipore, Bedford, MA, USA) were used in RIP assays. LINC00052 and miR-532-3p were detected in the pool of purified RNAs.

### RNA pull-down

Cells were transfected with biotinylated miR-532-3p, biotinylated mutant miR-532-3p or biotinylated negative controls. Cell lysates were incubated with M-280 streptavidin magnetic beads (Invitrogen, San Diego, CA, USA). The bound RNAs purified by TRIzol were subjected to qRT-PCR.

### Statistical analysis

Data are presented as the mean ± SD. Statistical comparisons between quantitative variables were carried out using Student’s *t*-tests and one-way ANOVAs. Differences were considered significant when *p* < 0.05. Statistics were calculated using SPSS 22.0 (SPSS Inc, Chicago, IL, USA).
